# Conflicting ordinal and metric depth information interferes with depth matching

**DOI:** 10.1167/jov.26.5.11

**Published:** 2026-05-28

**Authors:** Domenic Au, Jonathan Tong, Robert S. Allison, Laurie M. Wilcox

**Affiliations:** 1 Department of Biology and Centre for Vision Research, York University, Toronto, Ontario, Canada; 2 Department of Psychology and Centre for Vision Research, York University, Toronto, Ontario, Canada; 3 Department of Computer Science and Centre for Vision Research, York University, Toronto, Ontario, Canada; 4 Department of Psychology and Centre for Vision Research, York University, Toronto, Ontario, Canada

**Keywords:** occlusion, binocular disparity, Bayesian cue combination, stereopsis, augmented reality

## Abstract

Here, we investigate the effects of conflicting metric (binocular disparity) and ordinal (occlusion) depth information. In these experiments a virtual letter “A” was presented using a Microsoft HoloLens 2 optical-see through augmented reality display and was superimposed on a physical occluding surface with variable transparency. Observers performed a depth matching task and, for comparison, performed the same task without the occluder. Our results show that, when the surface was absent, or the letter was rendered in front of the surface, the letter was accurately localized. However, when the letter was rendered beyond the surface, observers progressively underestimated its distance. The magnitude of this error was modulated by the opacity of the occluding surface, but persisted even when additional depth information was provided by retinal size cues. Our results are well modeled using a Bayesian cue combination model with a uniform prior for letter positions in front of the surface and a non-uniform prior with a Gaussian fall-off for letter positions beyond the surface. These findings underscore the strong influence of incorrect occlusion arrangements, capable of distorting perceived depth even when binocular disparity is available.

## Introduction

### Ordinal and metric depth cues

Under natural viewing conditions, the visual system relies on multiple sources of depth information to perceive and act in our three-dimensional world. Binocular disparity must be integrated with monocular cues such as occlusion, motion-parallax, and perspective to form a coherent percept of three-dimensional space (Howard & Rogers, 2012). Assuming that the distance to fixation is available, binocular disparity, which is the positional offset between the retinal images in each eye, can provide metric depth information (i.e., how much depth there is between two points) (Foley, 1980; Sousa, Brenner, & Smeets, 2010). In contrast, ordinal cues offer qualitative depth information, indicating which object is closer or farther, but not by how much. Examples of ordinal cues include occlusion, where one object partially blocks another, and configural cues, such as figure–ground segmentation based on shape (Burge, Peterson, & Palmer, 2005; Burge, Fowlkes, & Banks, 2010).

Traditionally, studies of depth cue combination have focused on metric depth cues such as binocular disparity and motion-parallax (Richards, 1985; Tittle & Braunstein, 1993; Johnston, Cumming, & Landy, 1994; Ernst & Banks, 2002; Hartle & Wilcox, 2021). However, less is known about how ordinal and metric depth cues are combined by the human visual system. In a recent study, we investigated how occlusion (an ordinal cue) and binocular disparity (a metric cue) combined to determine perceived depth (Au, Allison, Gunasekera, & Wilcox, 2025). Using an augmented reality (AR) display, participants were presented with a virtual target superimposed on a physical surface. The distance of the virtual target was varied relative to the surface and participants adjusted the depth of a virtual probe to match the perceived depth of the target. When the target appeared in front of the surface or when the surface was absent, there was no conflict between disparity and occlusion cues, and matching performance was very accurate. However, when the target was rendered behind the surface, but remained visibly superimposed, creating a conflict between the cues, participants systematically underestimated its distance. This pattern of underestimation occurred in both peripersonal (0.35–0.50 m) and extrapersonal space (0.9–1.5 m). It persisted even when a visually guided reaching task was used, showing that proprioceptive feedback does not mitigate the effect of the conflicting visual cues. These results demonstrate that conflicting occlusion cues have a robust impact on perceived depth, consistently biasing position judgments when they contradict metric depth information.

Other related work on depth perception from occlusion has shown that the appearance and positioning of an occluder can influence the perceived depth of partially occluded objects. Hou, Lu, Zhou, and Liu (2006) showed observers two vertically aligned bars at different depths defined by binocular disparity. A horizontal bar was placed between them, with its position manipulated to either function as an occluder or not. When the horizontal bar was positioned closer to the observer, it acted as an occluder, leading the vertical bars to appear amodally completed behind it. In contrast, when the horizontal bar was farther away, the vertical bars were perceived as separate, disconnected objects. Observers were asked to judge which of the two vertical bars appeared closer in depth. Thresholds were significantly elevated when the horizontal bar was in front of the vertical bars, demonstrating that occlusion can increase the difficulty of depth discrimination. Interactions between occlusion and binocular disparity cues have also been observed under conditions involving illusory occlusion. That is, when a binocular bar and monocular bar of different orientations intersect such that the crossing point falls within one eye’s blind spot, the visual system may perceptually fill in one bar in front of the other, creating an illusory occlusion. The binocular bar appeared more distant when it appeared to be occluded, and closer when it was not occluded, despite the fact that disparity was equivalent in the two viewing conditions (Smith, Ropar, & Allen, 2017; Chen, Denison, Whitney, & Maus, 2018).

Although the studies described show that occlusion affects the amount of perceived depth from stereopsis, the opposite is also true. That is, the interpretation of a surface is influenced by local contrast relationships and can be modulated by binocular disparity. T-junctions and X-junctions are examples of this where the perception of occlusion and transparency is affected by stereopsis. T-junctions occur when the contour, which defines the edge of one surface, ends abruptly against another, signaling that the terminating surface is behind and thus occluded by the other. For example, when a vertical and horizontal bar overlap without texture or perspective cues, relative binocular disparity between the bars can give rise to a perceived depth separation where the bar that is closer appears to occlude the other bar (Nakayama & Shimojo, 1992). X-junctions arise when two or more surfaces intersect, possibly indicating transparency where one surface overlaps another without completely blocking it from view. When occlusion signals that a central element is in front, but binocular disparity places it behind an overlapping surface, the visual system can resolve this conflict by interpreting the overlapping surface as partially transparent (Cavanagh, 1987; Watanabe & Cavanagh, 1993). For example, an incomplete rectangle partially surrounding the corner of a central square can be perceived as a transparent surface in front of the square. This occurs when relative disparity between the surrounding and central elements indicate that the surrounding rectangles are closer than the central square.

#### Disparity–occlusion cue conflicts in AR

The integration of occlusion and binocular disparity also plays a key role in modern AR displays. In see-through AR systems, virtual content is additively overlaid onto the real world, meaning it is always superimposed on some part of the environment. When virtual elements are in the same direction as nearby physical surfaces, either intentionally (e.g., conformal overlays) or unintentionally, conflicts may arise between occlusion and binocular disparity cues. That is, virtual objects intended to appear behind physical surfaces may signal inconsistent depth order (occlusion violations) because disparity signals place them farther, but occlusion suggests they are in front (Kruijff, Swan, & Feiner, 2010; Au et al., 2025).

Related work has shown that such conflicts affect perception and performance. For example, occlusion violations have been shown to distort depth judgments by causing both overestimation (Johnson, Edwards, Griffin, & Hawkes, 2004) and underestimation (Fischer, Rosenberg, Leuze, Hargreaves, & Daniel, 2023; Au et al., 2025) of the location of virtual objects. These distortions have been attributed to the combined effects of incorrect occlusion cues, viewing distance, and vergence–accommodation conflicts. The latter occurs when the disparity-defined distance is different from that of the display’s accommodative distance (Foley, 1980; Hoffman, Girshick, Akeley, & Banks, 2008; Swan, Singh, & Ellis, 2015; Singh, Ellis, & Swan, 2018; Spiegel & Erkelens, 2025). Incorrect occlusion has also been shown to impair user interaction, leading to reduced pointing accuracy, longer task completion times, and decreased user confidence (Li, Hu, Wang, Bowman, & Lee, 2021).

### Ordinal and metric depth cue integration

Our previous work established that disagreement between ordinal and metric depth cues can significantly distort perceived depth (Au et al., 2025). However, it remains unclear how the visual system integrates these two fundamentally different types of information. Traditional models of Bayesian cue combination require that all contributing cues be expressed in a common unit to allow for weighted averaging based on their relative reliability (Ernst & Banks, 2002; Knill & Pouget, 2004). Ordinal cues, which provide only relative depth order without precise quantitative information, pose a challenge because they lack the units required for direct integration with metric cues. One approach to this problem was proposed in the weak fusion model, where each cue is considered in isolation, and then they are averaged to produce a combined estimate for a given stimulus or scene. The cues are transformed into common units before combining them in a process called *promotion* (Maloney & Landy, 1989; Cutting, Bruno, Brady, & Moore, 1992; Landy, Maloney, Johnston, & Young, 1995). According to this approach, for an ordinal cue like occlusion to be integrated with a metric cue like disparity, it would need to be transformed into a common unit space so that the cues can be combined using weighted averaging. This is a challenge because the ordinal nature of occlusion does not typically provide continuous information that would allow for such a transformation.

Stevenson and Koerding (2009) proposed a method by which ordinal occlusion constraints (e.g., object A must be in front of object B) could be combined probabilistically with metric depth information using conditional likelihood functions. They proposed that perceived depth is truncated when distances are modeled beyond an occluder to avoid scenarios where a supposedly occluded object is mistakenly interpreted as being in front. Critically, this approach does not 1) require conversion of ordinal cues into metric units or 2) model conflicting occlusion and disparity where an object’s position, defined by disparity, is visible beyond a surface. Instead, occlusion is treated as a soft constraint where the range of plausible depth values is adjusted based on a known position of an occluder while preserving uncertainty and noise from the binocular disparity cue.

### Current study

In a previous study, we used opaque wooden real-world occluders and AR-generated targets (Au et al., 2025). Here, we systematically vary the opacity of a physical occluder to manipulate the strength of the occlusion conflict. We use AR to present the target at different binocular disparities and sizes. We then propose a Bayesian cue integration model that accounts for the combination of ordinal and metric depth information, in cases where the cues are consistent and in conflict.

If surface opacity has no effect on perceived depth, results should be comparable with previous work with a fully opaque surface (Au et al., 2025). If reducing opacity completely eliminates the impact of the cue conflict, perceived depth should scale with the veridical depth of the stimulus. Finally, if opacity modulates the strength of the occlusion conflict, we expect a graded change in which perceived depth approaches veridical depth as the surface becomes more transparent.

## Experiment 1

In Experiment 1, we investigated whether it was possible to systematically modulate the disruptive effects of occlusion on perceived depth by varying surface opacity. By changing opacity, we varied the strength of the occlusion signal and anticipated a corresponding change in its effect on the perceived location of the virtual letter.

### Methods

#### Observers

Twelve observers participated in Experiment 1, four of whom identified as male, ranging in age from 21 to 36 years with a mean age of 27.2 years. All observers had stereoacuity thresholds of at least 40 arcseconds as determined by the Randot stereoacuity test and normal or corrected-to-normal visual acuity. The study protocols were approved by the York University Human Participants Review Committee.

#### Apparatus

All virtual stimuli were presented using a Microsoft HoloLens 2 with a diagonal field of view of 52^o^ and a refresh rate of 60 Hz. Each observer’s interpupillary distance was measured using Microsoft’s Eye Calibration tool before the experiment. To minimize head movement and restrict the use of motion parallax, observers viewed the stimuli with their heads stabilized in a chinrest equipped with vertical support posts.

**Figure 1.**
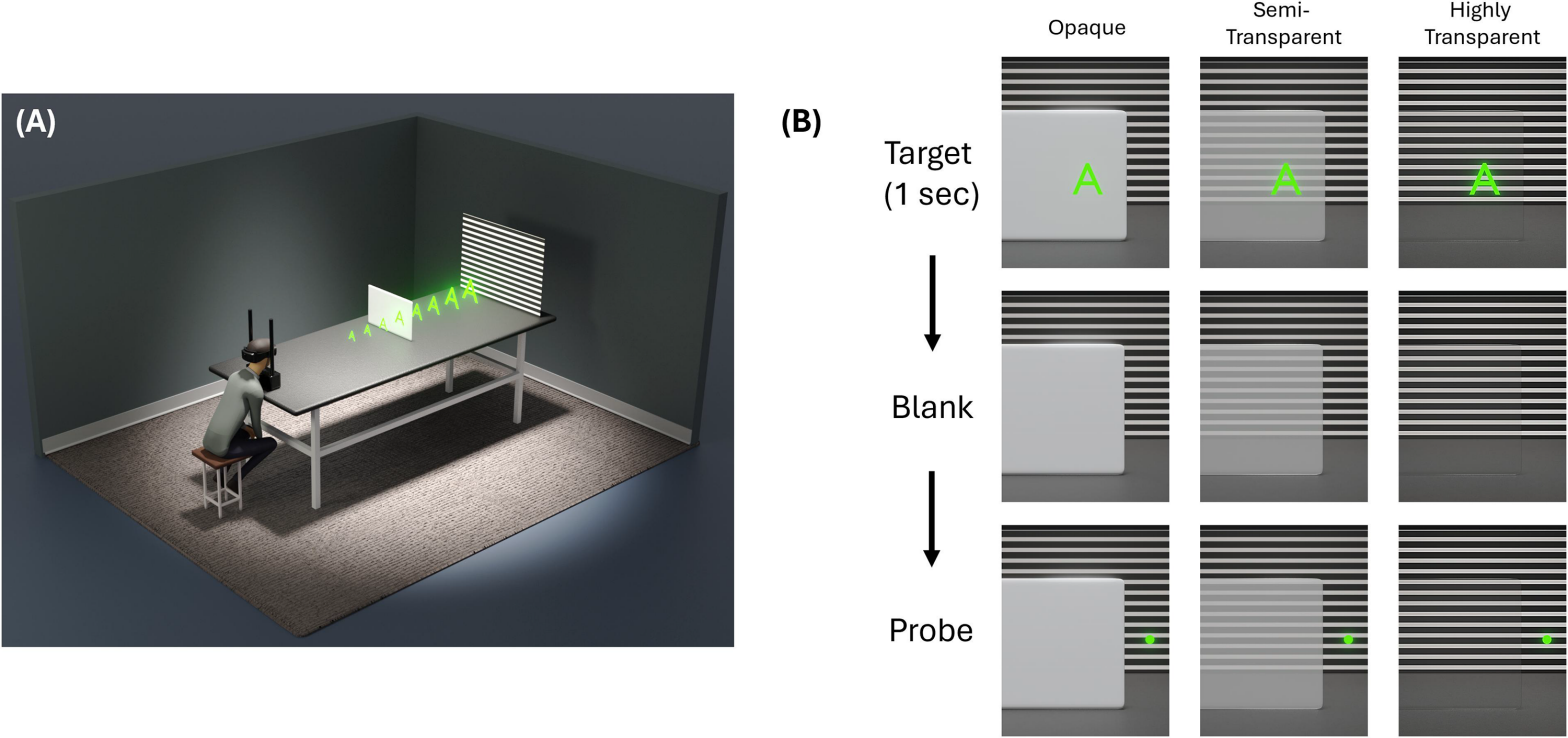
(A) Schematic of apparatus and stimulus used in Experiment 1. (B) Timeline for the trials demonstrating adjustment task for each surface opacity. A virtual letter is displayed at a given distance for 1 second, then disappears, followed by a circular virtual probe.

The physical environment consisted of an untextured test surface (28.5 × 21.0 cm) positioned at 1.2 m, frontoparallel to the observer along the midline on a black table. This test surface was a polymer dispersed liquid crystal film, which was capable of turning into a highly transparent state. A voltage regulator was used to adjust its opacity to three levels: opaque, semi-transparent, and highly transparent (Figure 1B). A background consisting of horizontal black and white lines was positioned at the end of the table, so the changes in transparency were clearly visible without providing horizontal disparity (Figure 1A). Surface opacity was quantified using Michelson contrast at each opacity level. Luminance measurements were made using a photometer of an sRGB BenQ XL2420Z display showing a central white rectangle (209.12 cd/m^2^) on a black background (1.24 cd/m^2^). The polymer dispersed liquid crystal surface was placed in front of the display, and luminance was recorded at each opacity level. Resulting Michelson contrasts were 0.001 (opaque), 0.62 (semi-transparent), 0.77 (highly transparent), and 0.98 (no surface).

#### Stimulus

**Figure 2.**
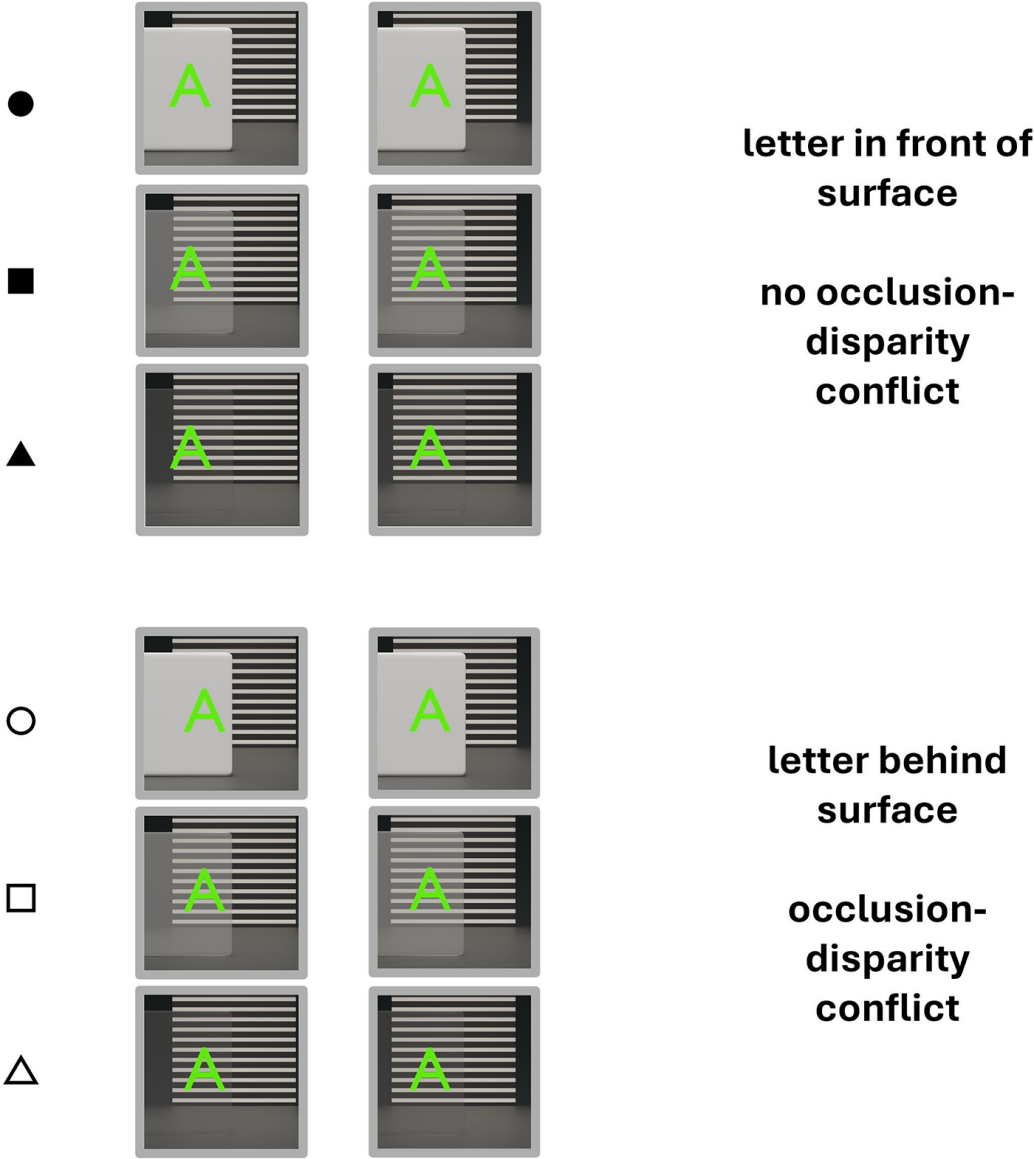
Example stereopairs arranged for cross fusion depicting stimuli used in Experiment 1. The top three stereopairs (solid symbols) show a non-cue conflict scenario with the letter positioned in front for each surface condition. Bottom three stereopairs (open symbols) show occlusion-disparity conflict where the letter is positioned behind the surface, but is still visible. The circle, square, and triangle symbols represent opaque, semi-transparent, and highly transparent surface conditions, respectively.

The virtual stimulus was a planar letter “A,” rendered using the Unity game engine and the Microsoft Mixed Reality Toolkit. The letter subtended a visual angle of 2.7^o^ in both height and width and was presented along the observer’s midline, superimposed on the surface (Figure 2). The letter’s distance was varied and its visual angle was held constant by scaling its size proportionally with distance. A baseline condition was also included in which the physical surface was removed, and only the letter was visible. A virtual circular probe (1 cm in diameter) was adjusted by observers to perform depth matches. The probe was positioned to the right of the surface to ensure that it did not overlap with the surface during the adjustment. To reduce color separation commonly observed in AR displays, a pure green shader material was applied to the letter and probe.

#### Procedure

A depth matching paradigm similar to that used by Swan, Singh, and Ellis (2015) was used. On each trial, observers were asked to match the position of the virtual probe to the remembered apparent distance of the virtual “A’ using a gamepad. The virtual letter was displayed for 1 second, followed by the probe. We chose a sequential presentation because we wanted to assess perceived depth. If the stimuli were presented simultaneously, observers could perform the task by minimizing the relative disparity between the target and probe (Foley, Applebaum, & Richards, 1975; Hartle & Wilcox, 2016). The absolute distance of the letter from the eyes ranged from 0.9 to 1.6 m in steps of 0.1 m with 10 trials per distance, which were tested in random order. The three levels of surface opacity were tested in separate blocks and counterbalanced across participants. The surface-absent condition was always tested last to prevent bias in depth matching during the surface present conditions, as viewing the surface absent condition first would reveal the true distances.

### Results

**Figure 3.**
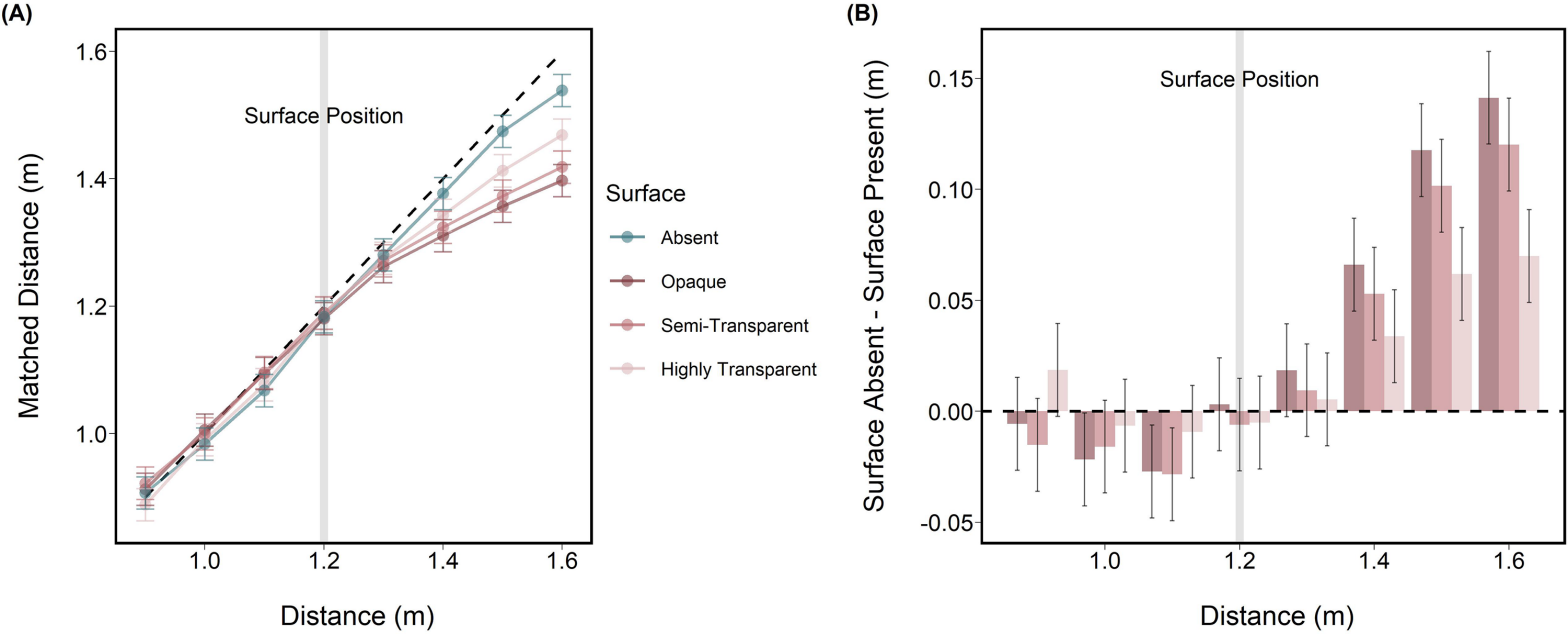
Experiment 1 results depicting (A) mean matched distance as a function of letter distance for different surface conditions. The black dashed line represents perfect performance according to the rendered disparity and (B) the signed difference between surface absent and each surface opacity plotted as a function of letter distance. In both plots, the vertical grey bar indicates the surface position and error bars show within-subject 95% confidence intervals.

Figure 3A shows observers’ matches as a function of letter distance with varying surface opacity. Matches were accurate for the no surface condition and for positions in front of the surface, irrespective of opacity. Matches for letters positioned beyond the surface were underestimated, with errors becoming progressively larger as the letter’s distance from the surface increased. The amount of underestimation varied with surface opacity, with the opaque surface producing the greatest underestimation, followed by the semi-transparent surface, and least for the highly transparent surface. Although accuracy improved in the highly transparent condition, distances beyond the surface were still consistently underestimated. A linear mixed-effects model with maximum likelihood estimation and a likelihood ratio χ^2^ test was used to assess the significance of fixed effects. There were significant effects of target distance χ^2^(7) = 5607.02, *p* < 0.0001, the surface condition, including surface absent and each opacity level, χ^2^(3) = 9.981, *p* < 0.0001, and their interaction, χ^2^(3) = 26.655, *p* < 0.0001. To assess the impact of surface opacity on matching accuracy, planned contrasts were run between the three surface opacity conditions. Significant differences were observed between all opacity conditions, with the exception of opaque and semi-transparent (Table 1).

**Table 1.** Contrast comparisons between surface opacity conditions. Bolded entries represent statistically significant differences.

Condition compared	*t*	*df*	*p* value
**Opaque – Surface absent**	**−8.757**	**365**	**<0.0001**
**Opaque – Highly transparent**	**4.956**	**365**	**<0.0001**
Opaque – Semi-transparent	−1.111	365	0.2671
**Semi-transparent – Surface absent**	**−7.645**	**365**	**<0.0001**
**Semi-transparent – Highly transparent**	**3.845**	**365**	**0.0001**
**Highly transparent – Surface absent**	**−3.800**	**365**	**0.0002**

When the letter was positioned behind the surface, the perceived target location was underestimated. To evaluate the extent the role of uncertainty in depth matches to this trend, a second linear mixed-effects model was conducted to assess whether within-subject variability of depth matches increased when the letter was behind the surface. The model was fit with observers’ standard deviations as the outcome variable for each target distance and surface condition. No significant increases in depth matching variability were observed with target distance, except for the highly transparent condition where increased variability was marginally significant (*p* = 0.048). Furthermore, to visualize within-subject variability, 95% within-subject confidence intervals are plotted in Figure 3. This approach removes between-subject variance, such that the intervals reflect only the subject and surface condition interaction (Loftus & Masson, 1994).

To more directly evaluate matching accuracy, we compared the slopes of the depth matching functions for distances in front vs behind the surface. Linear regressions were individually fit to distances 0.9 to 1.2 m (in front of the surface) and 1.3 to 1.6 m (behind the surface). No significant differences in slope were found between the in-front-of-surface and no-surface conditions. Likewise, surface opacity had no significant effect on slopes for positions behind the surface. Significant differences were only seen when comparing slopes for distances in front of the surface with those beyond it (Appendix Table A1).

The results of Experiment 1 replicate those of our previous study (Au et al., 2025) in showing that conflicting occlusion information produced systematic errors in depth matching. Matches were accurate when no surface was present or when targets appeared in front of the surface (no cue conflict) but the position of targets behind the surface was consistently underestimated (cue conflict). Importantly, the degree of underestimation varied with surface opacity, suggesting that the strength of the occlusion conflict influenced the amount of perceived depth. However, in this study, to isolate depth from binocular disparity, the letter was scaled with distance to avoid potentially informative retinal size cues. It is possible that the conflict between binocular disparity and size contributed to the pattern of underestimation seen here. We assess this in Experiment 2 by allowing retinal size to vary appropriately with the portrayed distance.

## Experiment 2

To evaluate the effect of the conflict between disparity and size in Experiment 1 on the observed underestimation, we repeated the study with a constant physical letter size and compared the results with Experiment 1.

### Methods

#### Observers

Eight observers participated in Experiment 2, ranging in age from 21 to 35 years with a mean age of 26.75 years. Two identified as male. All observers had previously participated in Experiment 1.

#### Apparatus

The physical environment and AR display in Experiment 2 were the same as described in Experiment 1, except that in Experiment 2, only the opaque, highly transparent, and surface absent conditions were tested.

#### Stimulus

**Figure 4.**
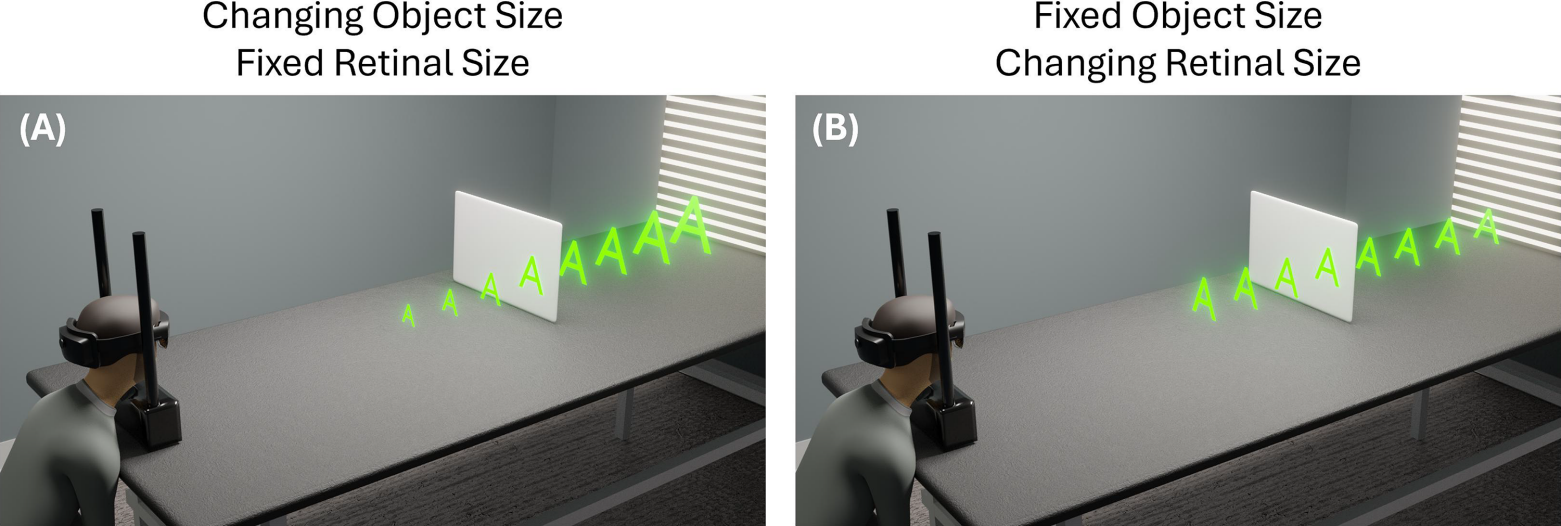
Schematic of virtual stimulus with (A) constant visual angle (Experiment 1) and (B) scaled visual angle with distance (Experiment 2).

The virtual stimulus consisted of the virtual letter “A.” Unlike Experiment 1, where the retinal size remained constant across distance, here, the angular size of the letter changed with distance. Consequently, the physical dimensions (height and width) of the letter were fixed at 5.65 cm. Angular sizes ranged from 3.6 to 2.0^o^ in accordance with distances of 0.9 to 1.6 m (Figure 4B).

#### Procedure

The same depth matching paradigm described in Experiment 1 was used here; again, the surface absent condition was completed last. Opaque and highly transparent surface conditions were tested in counterbalanced order.

### Results

**Figure 5.**
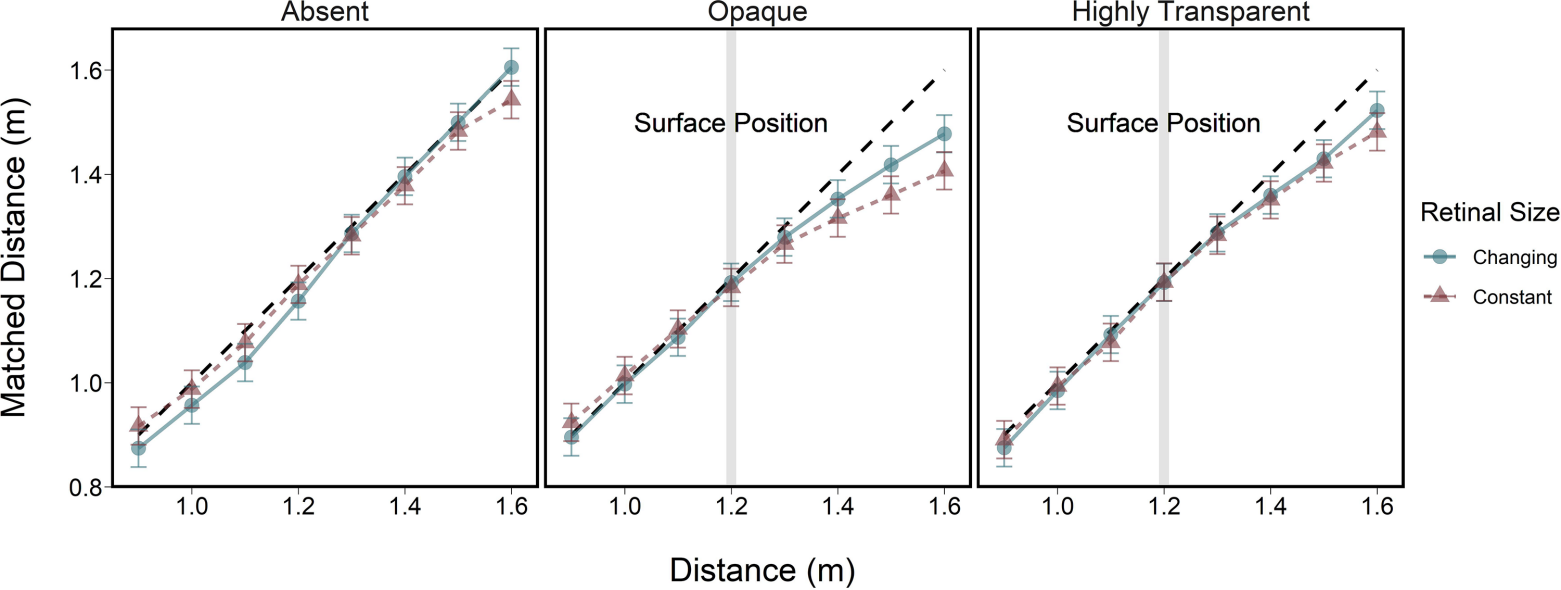
Results from Experiment 1 (red triangles) and Experiment 2 (blue circles), showing mean matched distance as a function of letter distance for constant and changing retinal size conditions. Each plot shows different surface conditions. The black dashed line represents perfect performance according to the rendered disparity and the vertical grey bar indicates the surface position and error bars show within-subject 95% confidence intervals.

Figure 5 shows observers’ distance matches plotted against portrayed letter distance as a function of surface opacity. The results from Experiment 1 are also shown for comparison. As found in Experiment 1, matches were accurate for the no surface condition and for distances in front of the surface regardless of opacity (Figure 5). No differences were found between the changing and fixed retinal sizes in the no surface and highly transparent surface conditions. However, when the surface was opaque, depth matching errors were generally smaller in the size-scaled condition.

A set of a linear mixed-effects model with maximum likelihood estimation and a likelihood ratio chi-square test was used to assess the significance of fixed effects. As expected, there were significant effects of target distance, χ^2^(7) = 5774.15, *p* < 0.0001, and surface type, χ^2^(2) = 7.279, *p* = 0.0008, but there was no effect of retinal size, χ^2^(1) = 1.197, *p* = 0.2747. There were significant interactions between target distance and surface type, χ^2^(2) = 34.692, *p* < 0.0001, and target distance and retinal size, χ^2^(1) = 23.953, *p* < 0.0001. No significant interactions between surface type and retinal size, χ^2^(2) = 1.335, *p* = 0.2644, or surface type, retinal size, and distance, χ^2^(2) = 1.562, *p* = 0.2112, were observed.

To evaluate the impact of retinal size on matching accuracy as a function of surface opacity, planned contrasts were run between specific surface and retinal size conditions. The contrasts were only implemented for target distances greater than the surface distance (1.3–1.6 m) to isolate distance conditions under the cue conflict. Matching was significantly more accurate for letters with changing retinal size compared to constant retinal size for the opaque surface, t(173) = 3.87, *p* = 0.0023. No significant differences were observed for either the highly transparent surface, *t*(173) = 1.38, *p* = 1.000, or the surface absent conditions, *t*(173) = 2.191, *p* = 0.4465.

In Experiment 2, we largely replicated the results of Experiment 1. That is, distance matching was accurate in the absence of cue conflicts, irrespective of surface opacity or the size of the target. However, when targets were positioned behind an opaque surface, depth judgments were slightly more accurate when retinal size varied naturally with distance compared with when it was held constant. This effect was not observed for the highly transparent surface, suggesting that size cues only improve matching accuracy under more extreme conflicts between disparity and occlusion.

## Cue integration model

To formalize the findings of Experiment 1 and to quantitatively model the integration of ordinal and metric depth cues, we implemented a Bayesian observer model. In this model, we assume a prior distribution for letter distance, given the occlusion cue of the letter being visible over the surface. This prior reflects the natural fact, based on physical material properties, that objects fully positioned behind an opaque surface, relative to the observer’s line of sight, will not be visible. In other words, under typical viewing, if a target is visible at all, it should appear in front of an opaque surface. By representing the ordinal nature of occlusion as a prior probability distribution, it becomes possible to meaningfully integrate it with metric depth cues such as binocular disparity. The proposed model was fit to individual observer’s results from Experiment 1. Because there was no main effect of retinal size in our second study, we did not include size as a depth cue in the model.

###  

#### Perceived surface distance

Although the physical surface was fixed at 1.2 m, to construct the occlusion prior we needed to take into account individual variability in its perceived location, for each level of opacity. We modeled the observer’s uncertainty in perceived surface location using a Gaussian distribution. Each observer estimated the position of the surface using an adjustable probe and the mean and standard deviation were used to model the distribution of the perceived surface localization for each opacity condition. Therefore, perceived surface location was modeled as a function of surface opacity:

(1) $$\begin{array}{c} \;\begin{array}{ccc} & & \mu_{\text{surf}(opacity)}=\text{mean}\;\text{perceived}\;\text{surface}\;\text{location}\\ & & \sigma_{\text{surf}(opacity)}=\text{standard}\;\text{deviation}\;\text{perceived}\\ & & \;\;\;\;\text{surface}\;\text{localization}\\ & & \mathrm{surface}\_\mathrm{dist}(x|opacity)\\ & & \;=N(x;\mu_{\text{surf}(opacity)},\sigma_{\text{surf}(opacity)}), \end{array} \end{array}$$

where N(x;μ,σ) is the normal distribution:

(2) $$N\left(x;\mu ,\sigma \right)=\frac{1}{\sqrt{2\pi \sigma^{2}}}\exp\left(-\frac{{\left(x-\mu \right)}^{2}}{2\sigma^{2}}\right)$$

#### Occlusion prior

We evaluated the probability of the letter being visible given its position relative to the surface *u*_*l*_(*x*; σ_opacity_) as follows.

**Figure 6.**
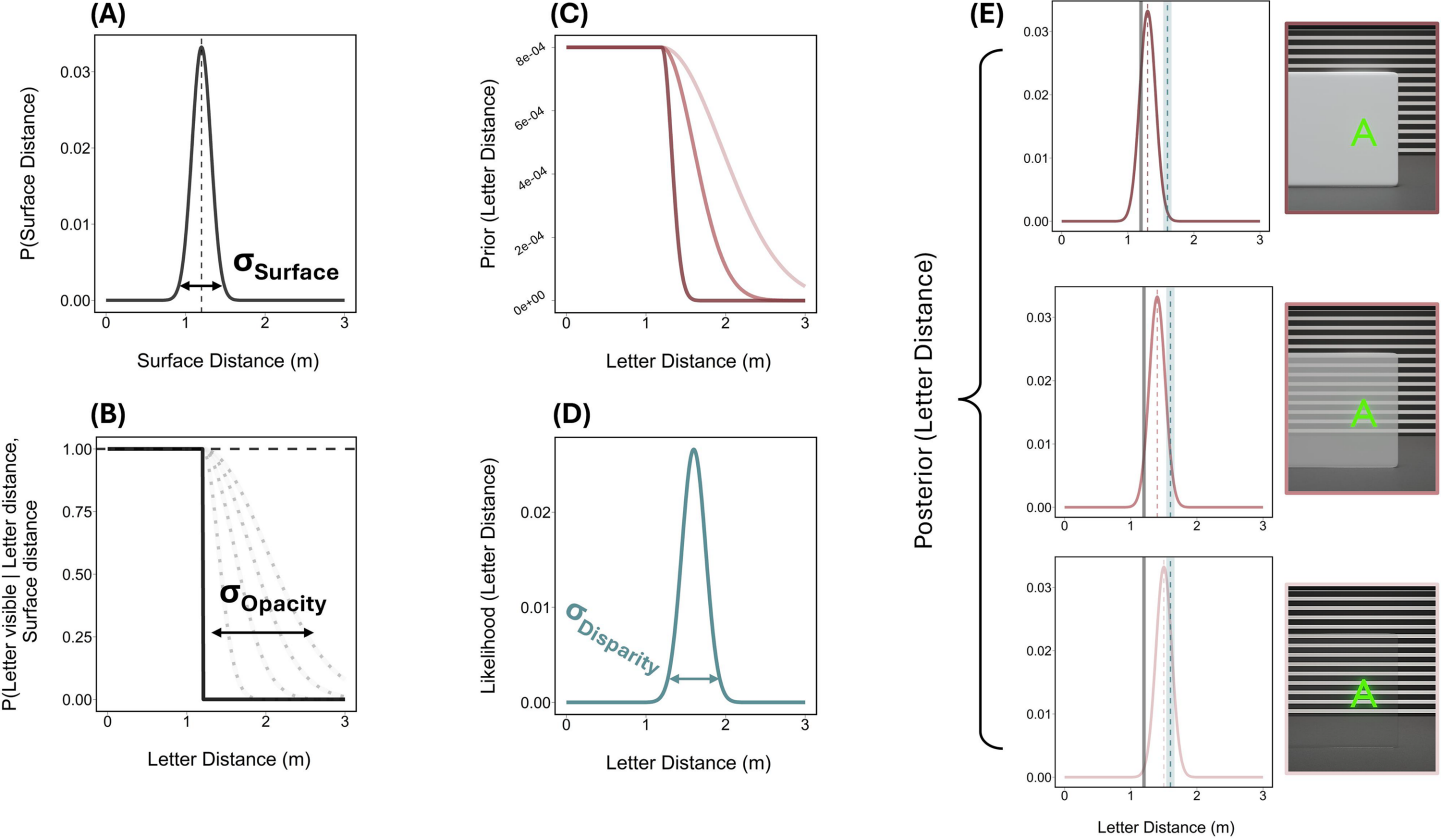
Workflow of cue integration model. (A) Distribution of perceived surface distance (σ_Surface_) is combined with (B) probability distribution of letter visibility given possible letter positions and surface opacity (σ_Opacity_). This forms (C) the occlusion prior for opaque, semi-transparent, and highly transparent surface conditions (dark to light pink curves) and is multiplied by (D) the likelihood for letter distance fit with the no surface condition (σ_Disparity_). (E) The peak of the resulting posterior for each surface opacity was used as model’s prediction for perceived letter distance relative to the true distance depicted by the vertical blue line and the surface position represented by the vertical grey bar.

(1) For each possible letter position, *x*, and the surface position, *x*_surf_, we defined an asymmetric probability distribution where σ_opacity_ was treated as a free parameter with smaller values yielding a steeper slope and larger values allowing an increased probability of letter visibility beyond the surface:
  (3) $$\;u_{l}\left(x;\sigma_{\text{opacity}}\right)=\left\{\begin{array}{cc} 1, & \text{if}\;x\leq x_{\text{surf}}\\ \frac{N(x;x_{\text{surf}},\sigma_{\text{opacity}})}{N(x_{\text{surf}};x_{\text{surf}},\sigma_{\text{opacity}})}, & \text{if}\;x>x_{\text{surf}} \end{array}\right.$$
  This function is flat (equal to 1) for values closer than *x*_surf_ and falls to 0 beyond *x*_surf_, resembling a step function before the slope is modulated by the σ_opacity_ parameter. The denominator ensures that the Gaussian is normalized so that the function has a peak of 1 at the surface position (Figure 6B).
(2) The resulting prior was constructed as a weighted sum of the probability that the surface is perceived at a given location and the probability that the letter would be visible from that location (Figure 6C):
  (4) $$\begin{array}{ccc} \; & & {\text{prior}}_{o}\left(x\right)=\sum_{l}\mathrm{surface}\_\mathrm{dist}\left(x_{\text{surf}}|opacity\right)\\ & & \;\cdot u_{l}\left(x,\sigma_{\text{opacity}}\right). \end{array}$$

The occlusion prior combines the perceived surface distance with an asymmetric distribution that is modulated by surface opacity. Although the perceived surface distance is fit using sensory information from the observer, we treat the resulting distribution (Equation 2) as an occlusion prior because it reflects expectations about depth conditioned on the perceived scene layout.

#### Binocular disparity

Distance matching results from the no surface baseline condition were used to model the weight of the binocular disparity cue. One-sample *t*-tests with Bonferroni correction were conducted to evaluate whether observers’ matches differed from the true distance of the letter. Across most distances (0.9–1.5 m), observers' matches were not significantly different from the veridical distance. Matches were only underestimated (*p* = 0.018) at the furthest distance (1.6 m), demonstrating a high degree of accuracy on average. From this we assume that the absence of systematic bias in the no surface condition suggests no evidence for the influence of a prior, allowing responses in this condition to be modeled primarily as arising from the sensory likelihood. Under this assumption, the variability of repeated matches reflects estimator variance, rather than the likelihood variance itself. To relate this variability to sensory uncertainty, we assume an approximately unbiased observer performing near optimal in this condition. Accordingly, a likelihood function was fit for each observer with a mean matched distance (μ_disp_) and standard deviation (σ_disp_) specific to each tested letter distance as follows (Figure 6D):

(5) $$\begin{array}{ccc} & & \mu_{\text{disp}}=\text{mean}\;\text{matched}\;\text{distance}\;\text{(no}\;\text{surface)}\\ & & \sigma_{\text{disp}}=\text{standard}\;\text{deviation}\;\text{(no}\;\text{surface)}\\ & & {\text{likelihood}}_{\text{disp}}\left(x\right)=N\left(x;\mu_{\text{disp}},\sigma_{\text{disp}}\right). \end{array}$$

#### Posterior prediction

The likelihood function for binocular disparity was multiplied with the constructed prior to produce the posterior distribution (Figure 6E). The mode of the posterior was used as the model’s prediction for the depth match. The effect of surface opacity was captured by the parameter, σ_opacity_, which influenced the predicted depth matches relative to the true position defined by binocular disparity. For example, with a disparity-defined letter distance of 1.6 m, the model predicted that the perceived depth would be 1.4 m (0.2 m closer than it actually is) in the presence of a conflicting fully opaque surface. Typically, greater opacity resulted in larger deviations from veridical depth matching, with opaque, semi-transparent, and highly transparent surfaces producing large, moderate, and minimal predicted offsets, respectively:

(6) $$\text{posterior}\left(x\right)\propto {\text{likelihood}}_{\text{disp}}\left(x\right)\cdot {\text{prior}}_{o}\left(x\right).$$

#### Model performance

**Figure 7.**
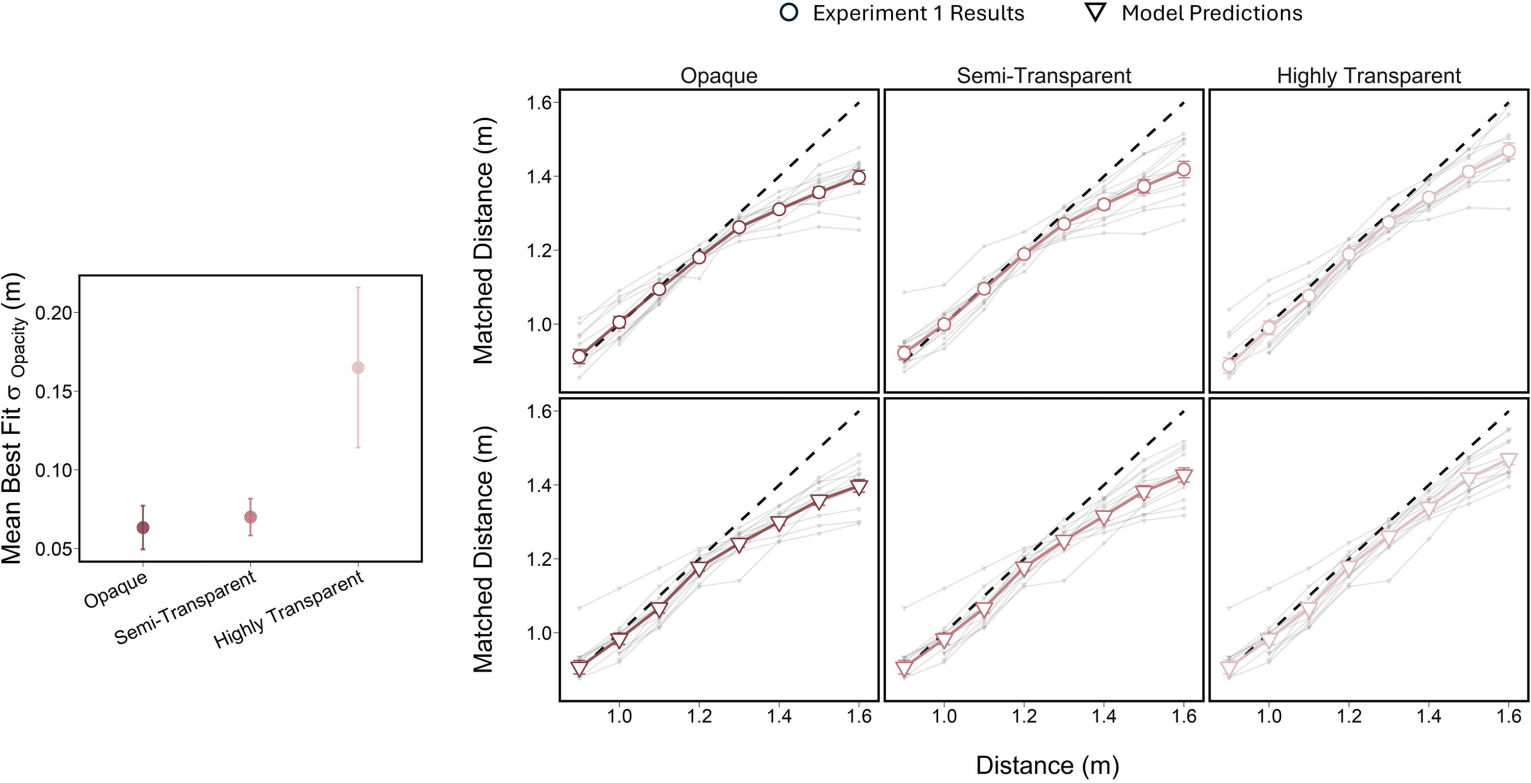
Plot on the left shows the mean best fit σ_opacity_ parameter for each surface opacity. Error bars represent standard error of the mean. Plots on the right show Experiment 1 results (top row of plots with open circles) and mean model predictions (bottom row of plots with open triangles) for each surface condition. Light gray points represent individual observers’ results for Experiment 1 and the respective model fits.

Each observer’s matching results were modeled individually. As shown in Figure 7, overall, the model successfully captured the general pattern of results from Experiment 1. That is, the model and Experiment 1’s results showed the largest underestimates in the fully opaque condition, with matching accuracy improving as surface transparency increased. The model also reproduced the accurate depth matches for targets positioned in front of the occluder and the systematic underestimation of targets behind it, with the magnitude of this bias modulated by the occluder’s opacity (Figure 7). This was reflected in the mean best fit σ_opacity_ values for the different surface opacities. The goodness of model fits varied slightly between observers, but overall the predictions generally conformed with Experiment 1 results (Appendix Table A2).

To assess the model’s performance, a linear mixed-effects model was fit with surface opacity as a fixed effect and observers as random factors. There was a significant effect of surface opacity on the slope of the occlusion prior, σ_opacity_, χ^2^(2) = 4.889, *p* = 0.0175. Planned contrasts revealed that the σ_opacity_ parameter was significantly larger for the highly transparent surface condition compared to the opaque, *t*(22) = 2.795, *p* = 0.0317, and semi-transparent, *t*(22) = 2.612, *p* = 0.0478, surface conditions, demonstrating that a shallower slope reflects an occlusion prior with persisting target visibility beyond the surface. No significant difference was observed between the opaque and semi-transparent conditions.

## Discussion

The primary objective of this study was to evaluate whether the effects of conflicting occlusion and binocular disparity would vary systematically with the strength of the occlusion signal (i.e., surface opacity), and the extent to which conflicting size contributed to underestimates. A second goal was to evaluate whether the psychophysical outcomes could be effectively modeled using a Bayesian framework.

### Surface opacity and occlusion cue strength

In Experiment 1, we examined the influence of conflicting occlusion cues on depth localization across three levels of surface opacity: fully opaque, semi-transparent, and highly transparent. The rationale was that increased surface transparency makes it more ecologically plausible for an object to be visible through the surface, thereby reducing the degree of conflict between the occlusion signal and binocular disparity. The results showed that depth matching performance improved with increased transparency. This finding aligns with previous studies that have demonstrated that transparent or semi-transparent objects provide useful visual information that can aid in interpreting relative depth and object ordering (Zhai, Buxton, & Milgram, 1996; Hillstrom, Wakefield, & Scholey, 2013; Ping et al., 2020).

Interestingly, despite this improvement, position in depth remained underestimated in the highly transparent surface condition for positions further than the occluding surface (i.e., in the conflict conditions). It should be noted that a clear surface edge boundary was visible even for the highly transparent case, signaling the presence of a surface. Edge contours have been shown to be potent cues for relative depth and figure–ground segmentation where the region with the visible edge is perceived as closer (Palmer & Ghose, 2008), which would reinforce the existence of an occluder.

Another potential explanation for the systematic underestimation in Experiment 1 is that, under natural viewing conditions, a surface with varying opacity would reduce the luminance of objects viewed behind it. The amount of luminance reduction would depend on the level of transparency, but in our experiment, the virtual letter’s contrast and luminance remained unchanged when rendered behind the surface. Because contrast is a pictorial depth cue, where high-contrast elements tend to be perceived as nearer (Farnè, 1977; O’Shea, Blackburn, & Ono, 1994; Singh, Ellis, & Swan, 2018), this lack of contrast attenuation may have led observers to perceive the target as being closer than its intended depth. However, we note that this situation is most representative of typical AR use cases.

### Relative size cues

In our second study, size varied linearly with distance. This was done to evaluate the impact of the conflict between disparity and size from Experiment 1, where letter size was scaled to maintain a constant angular size. Interestingly, the results revealed no overall main effect of retinal size on matching accuracy. Improvements occurred only under the strongest occlusion conflict conditions. That is, the most distant target position behind an opaque occluder. It has been shown that, when binocular disparity is available, observers may rely less on other monocular depth cues. For instance, when viewing objects with depth defined by motion-parallax and binocular disparity, observers disregard the less reliable motion-parallax cue and rely primarily on binocular disparity for estimating depth (Bülthoff & Mallot, 1988; Hartle & Wilcox, 2021). Therefore, it is possible that, in the presence of binocular disparity, retinal size cues typically do not contribute sufficient depth information to disambiguate distortions in perceived depth from conflicting occlusion. Also, in our study, virtual letters were presented successively. Typically, relative size cues are most effective when multiple objects are viewed at different distances simultaneously. In a more cue-rich environment with strong perspective and texture gradient cues, size cues may be more salient (Diaz, Walker, Szafir, & Szafir, 2017; Hornsey & Hibbard, 2021; Adams, Stefanucci, Creem-Regehr, & Bodenheimer, 2022; Yildiz et al., 2024). In particular, familiar size, which is from prior knowledge of an object’s size, can be used to infer distance. It has been shown that familiar size can affect an object’s perceived size and distance, thereby strengthening the potential impact of angular size varying with distance (Bolles & Bailey, 1956; Maltz et al., 2021; Rzepka et al., 2023).

In Experiments 1 and 2, overall the position of the letter was underestimated in the surface-absent conditions. Introducing additional objects in a scene, with distances defined by binocular disparity, improves distance estimation (Sousa, Brenner, & Smeets, 2010). Therefore, the slight underestimation in the surface absent conditions is likely a consequence of the lack of a reference surface.

### Integration of ordinal and metric depth cues

Our Bayesian observer model demonstrates that occlusion, an ordinal depth cue, can be effectively integrated with metric depth cues like binocular disparity when modeled as a prior expectation about where an object is likely to be visible relative to an occluding surface. This approach differs from the weak fusion model, where cues are promoted into a common unit space before combining them (Maloney & Landy, 1989; Cutting et al., 1992; Landy et al., 1995). Rather than transforming the occlusion cue into metric space, our model represents it as a prior on target position given target visibility, which is modulated by the strength (surface opacity) of the cue. Furthermore, our model enables us to capture cue conflicts between occlusion and binocular disparity. Previous work that has modeled occlusion with ecologically invalid configurations predicted perceived depth at a fixed position (Stevenson & Koerding, 2009). For example, if an object’s distance exceeds that of an occluder, the perceived distance will be truncated to the distance of the occluder. In contrast, in our studies, observers were able to match positions beyond the occluding surface, and the model was constructed to include these cue conflict scenarios. This finding suggests that, rather than a hard limit on perceived distance beyond an occluder, the visual system relies on an asymmetric occlusion prior that favors visibility of objects in front of a surface while there is a reduction in the likelihood of object visibility behind it. Additionally, according to our model, when a surface becomes more transparent, the occlusion prior becomes shallower, reflecting a relaxed expectation about occlusion, thus allowing for greater tolerance to conflicting occlusion and disparity cues. This modulation of the prior by opacity provides a principled way to capture different surface or object materials in real-world contexts, where traditional depth cues may be in conflict or degraded.

## Conclusions

We examined how occlusion and binocular disparity interact to influence perceived depth. These results highlight the impact of occlusion on depth localization where, even in the presence of metric depth cues like binocular disparity and relative size, incorrect occlusion consistently biases the perceived target distance to be closer than its true rendered location. Our results also show that the occlusion cue can be modeled as a prior for expected object visibility that varies in strength with surface opacity. This framework allows for ordinal and metric depth cue integration, enabling predictions in perceived object location under incorrect occlusion. Such a model formulation offers a flexible basis for potentially extending this approach to other surface characteristics, including surface texture, contrast, and scene complexity with multiple objects. Finally, in addition to providing insight into the combination of ordinal and metric depth cues, these results highlight the importance of accurately rendering occlusion arrangements in AR environments.

## Appendix

**Table A1.** Experiment 1 summary of pairwise comparisons of regression slopes between surface conditions for binned distance ranges. “Closer” refers to distances of 0.9 to 1.2 m while “Further” is 1.3 to 1.6 m. Bolded entries refer to statistical significance for the *t*-values after Bonferroni correction. A *p* value < 0.05 and < 0.01 is indicated by * and **, respectively. Shaded cells highlight comparisons between surface opacities for distance ranges further than the surface.

	Reference condition, pairwise difference, *t*_11_ =						
Condition compared	*HT*_*close*_	*ST*_*far*_	*ST*_*close*_	*O*_*far*_	*O*_*close*_	*SA*_*far*_	*SA*_*close*_
Highly transparent further (*HT*_*far*_)	**−6.59****	3.02	**−4.74***	3.16	−3.35	−3.96	**−6.05****
Highly transparent closer (*HT*_*close*_)	–	**8.23****	2.09	**9.36****	2.00	1.93	1.72
Semi-transparent further (*ST*_*far*_)	–	–	**−7.87****	0.86	**−5.35****	**−7.25****	**−8.41****
Semi-transparent closer (*ST*_*close*_)	–	–	–	**7.63****	0.09	0.51	−0.41
Opaque further (*O*_*far*_)	–	–	–	–	**−6.71****	**−6.98****	**−8.45****
Opaque closer (*O*_*close*_)	–	–	–	–	–	0.25	−0.33
Surface absent further (*SA*_*far*_)	–	–	–	–	–	–	−0.81
Surface absent closer (*SA*_*close*_)	–	–	–	–	–	–	–

**Table A2.** Cue integration model fitting results by observer and surface opacity. Root mean square error (RMSE) and σ_opacity_ values are shown separately for each surface opacity condition across all observers. The final column shows the mean ± standard error across (SE) observers.

	Observer												
Condition	1	2	3	4	5	6	7	8	9	10	11	12	Mean ± SE
RMSE													
Opaque	0.033	0.025	0.041	0.047	0.045	0.021	0.026	0.041	0.142	0.065	0.030	0.034	0.046 ± 0.009
Semi-transparent	0.040	0.029	0.040	0.081	0.056	0.028	0.034	0.039	0.090	0.029	0.026	0.034	0.044 ± 0.006
Highly transparent	0.054	0.068	0.037	0.074	0.036	0.048	0.059	0.018	0.092	0.052	0.031	0.021	0.049 ± 0.006
$\sigma_{\text{opacity}}$													
Opaque	0.08	0.02	0.06	0.02	0.02	0.08	0.06	0.06	0.20	0.04	0.06	0.06	0.063 ± 0.014
Semi-transparent	0.06	0.02	0.14	0.02	0.02	0.08	0.06	0.08	0.14	0.08	0.06	0.08	0.070 ± 0.012
Highly transparent	0.10	0.06	0.08	0.02	0.04	0.44	0.06	0.12	0.48	0.08	0.06	0.44	0.165 ± 0.051

## References

[bib1] Adams H., Stefanucci J., Creem-Regehr S., & Bodenheimer B. (2022). Depth perception in augmented reality: The effects of display, shadow, and position. In *2022 IEEE Conference on Virtual Reality and 3D User Interfaces (VR)* (pp. 792–801).

[bib2] Au D., Allison R. S., Gunasekera I., & Wilcox L. M. (2025). Misperception of the distance of virtual augmentations. In *2025 IEEE Conference Virtual Reality and 3D User Interfaces (VR)* (pp. 516–525).

[bib3] Bolles R. C., & Bailey D. E. (1956). Importance of object recognition in size constancy. *Journal of Experimental Psychology,* 51(3), 222. 13306868 10.1037/h0048080

[bib4] Bülthoff H. H., & Mallot H. A. (1988). Integration of depth modules: Stereo and shading. *Journal of the Optical Society of America A,* 5(10), 1749–1758. 10.1364/josaa.5.001749 3204438

[bib5] Burge J., Fowlkes C. C., & Banks M. S. (2010). Natural-scene statistics predict how the figure–ground cue of convexity affects human depth perception. *Journal of Neuroscience,* 30(21), 7269–7280. 20505093 10.1523/JNEUROSCI.5551-09.2010 PMC3062505

[bib6] Burge J., Peterson M. A., & Palmer S. E. (2005). Ordinal configural cues combine with metric disparity in depth perception. *Journal of Vision,* 5(6), 5, 10.1167/5.6.5. 16097866

[bib7] Cavanagh P. (1987). Reconstructing the third dimension: Interactions between color, texture, motion, binocular disparity, and shape. *Computer Vision, Graphics, and Image Processing,* 37(2), 171–195.

[bib8] Chen Z., Denison R. N., Whitney D., & Maus G. W. (2018). Illusory occlusion affects stereoscopic depth perception. *Scientific Reports,* 8(1), 5297. 29593236 10.1038/s41598-018-23548-3 PMC5871781

[bib9] Cutting J. E., Bruno N., Brady N. P., & Moore C. (1992). Selectivity, scope, and simplicity of models: A lesson from fitting judgments of perceived depth. *Journal of Experimental Psychology: General,* 121(3), 364. 1402706 10.1037//0096-3445.121.3.364

[bib10] Diaz C., Walker M., Szafir D. A., & Szafir D. (2017). Designing for depth perceptions in augmented reality. In *2017 IEEE International Symposium on Mixed and Augmented Reality (ISMAR)* (pp. 111–122).

[bib11] Ernst M. O., & Banks M. S. (2002). Humans integrate visual and haptic information in a statistically optimal fashion. *Nature,* 415(6870), 429–433. 11807554 10.1038/415429a

[bib12] Farnè M. (1977). Brightness as an indicator to distance: Relative brightness per se or contrast with the background? *Perception,* 6(3), 287–293. 866085 10.1068/p060287

[bib13] Fischer M., Rosenberg J., Leuze C., Hargreaves B., & Daniel B. (2023). The impact of occlusion on depth perception at arm's length. *IEEE Transactions on Visualization and Computer Graphics,* 29(11), 4494–4502. 37782607 10.1109/TVCG.2023.3320239

[bib14] Foley J. (1980). Binocular distance perception. *Psychological Review,* 87(5), 411. 7413886

[bib15] Foley J., Applebaum T., & Richards W. (1975). Stereopsis with large disparities: Discrimination and depth magnitude. *Vision Research,* 15(3), 417–421. 1136159 10.1016/0042-6989(75)90091-7

[bib16] Hartle B., & Wilcox L. M. (2016). Depth magnitude from stereopsis: Assessment techniques and the role of experience. *Vision Research,* 125, 64–75. 27369096 10.1016/j.visres.2016.05.006

[bib17] Hartle B., & Wilcox L. M. (2021). Cue vetoing in depth estimation: Physical and virtual stimuli. *Vision Research,* 188, 51–64. 34289419 10.1016/j.visres.2021.07.003

[bib18] Hillstrom A. P., Wakefield H., & Scholey H. (2013). The effect of transparency on recognition of overlapping objects. *Journal of Experimental Psychology: Applied,* 19(2), 158. 23795982 10.1037/a0033367

[bib19] Hoffman D. M., Girshick A. R., Akeley K., & Banks M. S. (2008). Vergence–accommodation conflicts hinder visual performance and cause visual fatigue. *Journal of Vision,* 8(3), 33, 10.1167/8.3.33. PMC2879326 18484839

[bib20] Hornsey R. L., & Hibbard P. B. (2021). Contributions of pictorial and binocular cues to the perception of distance in virtual reality. *Virtual Reality,* 25(4), 1087–1103.

[bib21] Hou F., Lu H., Zhou Y., & Liu Z. (2006). Amodal completion impairs stereoacuity discrimination. *Vision Research,* 46(13), 2061–2068. 16472836 10.1016/j.visres.2005.12.010

[bib22] Howard I. P., & Rogers B. J. (2012). *Perceiving in Depth, volume 2: Stereoscopic Vision* (No. 29). New York: Oxford University Press.

[bib23] Johnson L., Edwards P., Griffin L., & Hawkes D. (2004). Depth perception of stereo overlays in image-guided surgery. In: *Medical imaging 2004: Image perception, observer performance, and technology assessment* (Vol. 5372, pp. 263–272). Bellingham, WA: SPIE.

[bib24] Johnston E. B., Cumming B. G., & Landy M. S. (1994). Integration of stereopsis and motion shape cues. *Vision Research,* 34(17), 2259–2275. 7941420 10.1016/0042-6989(94)90106-6

[bib25] Knill D. C., & Pouget A. (2004). The Bayesian brain: The role of uncertainty in neural coding and computation. *Trends in Neurosciences,* 27(12), 712–719. 15541511 10.1016/j.tins.2004.10.007

[bib26] Kruijff E., Swan J. E., & Feiner S. (2010). Perceptual issues in augmented reality revisited. In: *2010 IEEE International Symposium on Mixed and Augmented Reality* (pp. 3–12). Sydney, Australia.

[bib27] Landy M. S., Maloney L. T., Johnston E. B., & Young M. (1995). Measurement and modeling of depth cue combination: In defense of weak fusion. *Vision Research,* 35(3), 389–412. 7892735 10.1016/0042-6989(94)00176-m

[bib28] Li Y., Hu D., Wang B., Bowman D. A., & Lee S. W. (2021). The effects of incorrect occlusion cues on the understanding of barehanded referencing in collaborative augmented reality. *Frontiers in Virtual Reality,* 2, 681585.

[bib29] Loftus G. R., & Masson M. E. (1994). Using confidence intervals in within-subject designs. *Psychonomic Bulletin & Review,* 1(4), 476–490. 24203555 10.3758/BF03210951

[bib30] Maloney L. T., & Landy M. S. (1989). A statistical framework for robust fusion of depth information. In: *Visual Communications and Image Processing IV* (Vol. 1199, pp. 1154–1163). Bellingham, WA: SPIE.

[bib31] Maltz M. V., Stubbs K. M., Quinlan D. J., Rzepka A. M., Martin J. R., & Culham J. C. (2021). Familiar size affects the perceived size and distance of real objects even with binocular vision. *Journal of Vision,* 21(10), 21, 10.1167/jov.21.10.21. PMC8479574 34581767

[bib32] Nakayama K., & Shimojo S. (1992). Experiencing and perceiving visual surfaces. *Science,* 257(5075), 1357–1363. 1529336 10.1126/science.1529336

[bib33] O'Shea R. P., Blackburn S. G., & Ono H. (1994). Contrast as a depth cue. *Vision Research,* 34(12), 1595–1604. 7941367 10.1016/0042-6989(94)90116-3

[bib34] Palmer S. E., & Ghose T. (2008). Extremal edge: A powerful cue to depth perception and figure-ground organization. *Psychological Science,* 19(1), 77–83. 18181795 10.1111/j.1467-9280.2008.02049.x

[bib35] Ping J., Thomas B. H., Baumeister J., Guo J., Weng D., & Liu Y. (2020). Effects of shading model and opacity on depth perception in optical see-through augmented reality. *Journal of the Society for Information Display,* 28(11), 892–904.

[bib36] Richards W. (1985). Structure from stereo and motion. *Journal of the Optical Society of America A,* 2(2), 343–349. 10.1364/josaa.2.000343 3973765

[bib37] Rzepka A.M., Hussey K. J., Maltz M. V., Babin K., Wilcox L.M., & Culham J. C. (2023). Familiar size affects perception differently in virtual reality and the real world. *Philosophical Transactions of the Royal Society B: Biological Sciences,* 378(1869), 20210464. 10.1098/rstb.2021.0464 PMC9745877 36511414

[bib38] Singh G., Ellis S. R., & Swan J. E. (2018). The effect of focal distance, age, and brightness on near-field augmented reality depth matching. *IEEE Transactions on Visualization and Computer Graphics,* 26(2), 1385–1398. 30222576 10.1109/TVCG.2018.2869729

[bib39] Smith D., Ropar D., & Allen H. A. (2017). The integration of occlusion and disparity information for judging depth in autism spectrum disorder. *Journal of Autism and Developmental Disorders,* 47, 3112–3124. 28688073 10.1007/s10803-017-3234-x PMC5602035

[bib40] Sousa R., Brenner E., & Smeets J. (2010). A new binocular cue for absolute distance: Disparity relative to the most distant structure. *Vision Research,* 50(18), 1786–1792. 20595037 10.1016/j.visres.2010.05.035

[bib41] Spiegel D., & Erkelens I. (2025). Stereopsis–occlusion conflicts impair visual performance in augmented reality. *Journal of the Society for Information Display,* 33(8), 937–947, 10.1002/jsid.2095.

[bib42] Stevenson I., & Koerding K. (2009). Structural inference affects depth perception in the context of potential occlusion. *Advances in Neural Information Processing Systems,* 22.

[bib43] Swan J. E., Singh G., & Ellis S. R. (2015). Matching and reaching depth judgments with real and augmented reality targets. *IEEE Transactions on Visualization and Computer Graphics,* 21(11), 1289–1298. 26340777 10.1109/TVCG.2015.2459895

[bib44] Tittle J. S., & Braunstein M. L. (1993). Recovery of 3-D shape from binocular disparity and structure from motion. *Perception & Psychophysics,* 54(2), 157–169. 8361830 10.3758/bf03211751

[bib45] Watanabe T., & Cavanagh P. (1993). Transparent surfaces defined by implicit × junctions. *Vision Research,* 33(16), 2339–2346. 8273298 10.1016/0042-6989(93)90111-9

[bib46] Yildiz G. Y., Skarbez R., Sperandio I., Chen S. J., Mulder I. J., & Chouinard P. A. (2024). Linear perspective cues have a greater effect on the perceptual rescaling of distant stimuli than textures in the virtual environment. *Attention, Perception, & Psychophysics,* 86(2), 653–665. 10.3758/s13414-023-02834-x 38182938

[bib47] Zhai S., Buxton W., Milgram P. (1996). The partial-occlusion effect: Utilizing semitransparency in 3D human-computer interaction. *ACM Transactions on Computer-Human Interaction (TOCHI),* 3(3), 254–284.

